# More Than Just Simple Interaction between STIM and Orai Proteins: CRAC Channel Function Enabled by a Network of Interactions with Regulatory Proteins

**DOI:** 10.3390/ijms22010471

**Published:** 2021-01-05

**Authors:** Sascha Berlansky, Christina Humer, Matthias Sallinger, Irene Frischauf

**Affiliations:** Institute of Biophysics, Johannes Kepler University, 4020 Linz, Austria; sascha.berlansky@jku.at (S.B.); christina.humer_1@jku.at (C.H.); matthias.sallinger@jku.at (M.S.)

**Keywords:** Ca^2+^, SOCE, CRAC, STIM1, Orai1, positive/negative modulators of CRAC channel activation, protein–protein interactions, indirect regulation, protein trafficking, ER-PM junctions, clustering, inactivation

## Abstract

The calcium-release-activated calcium (CRAC) channel, activated by the release of Ca^2+^ from the endoplasmic reticulum (ER), is critical for Ca^2+^ homeostasis and active signal transduction in a plethora of cell types. Spurred by the long-sought decryption of the molecular nature of the CRAC channel, considerable scientific effort has been devoted to gaining insights into functional and structural mechanisms underlying this signalling cascade. Key players in CRAC channel function are the Stromal interaction molecule 1 (STIM1) and Orai1. STIM1 proteins span through the membrane of the ER, are competent in sensing luminal Ca^2+^ concentration, and in turn, are responsible for relaying the signal of Ca^2+^ store-depletion to pore-forming Orai1 proteins in the plasma membrane. A direct interaction of STIM1 and Orai1 allows for the re-entry of Ca^2+^ from the extracellular space. Although much is already known about the structure, function, and interaction of STIM1 and Orai1, there is growing evidence that CRAC under physiological conditions is dependent on additional proteins to function properly. Several auxiliary proteins have been shown to regulate CRAC channel activity by means of direct interactions with STIM1 and/or Orai1, promoting or hindering Ca^2+^ influx in a mechanistically diverse manner. Various proteins have also been identified to exert a modulatory role on the CRAC signalling cascade although inherently lacking an affinity for both STIM1 and Orai1. Apart from ubiquitously expressed representatives, a subset of such regulatory mechanisms seems to allow for a cell-type-specific control of CRAC channel function, considering the rather restricted expression patterns of the specific proteins. Given the high functional and clinical relevance of both generic and cell-type-specific interacting networks, the following review shall provide a comprehensive summary of regulators of the multilayered CRAC channel signalling cascade. It also includes proteins expressed in a narrow spectrum of cells and tissues that are often disregarded in other reviews of similar topics.

## 1. Introduction

Alterations in the concentration of freely available cytosolic calcium (Ca^2+^) represent a vital element of cellular signal transmission, allowing for the release of inflammatory mediators, as well as of messengers like neurotransmitters or hormones, to relay information to distant target sites. On the single-cell level, Ca^2+^ transients are critical for the contraction of myocytes, the induction of changes in gene expression patterns, and cell proliferation and differentiation, in addition to driving apoptotic cell death [1,2].

Ca^2+^ is an outstanding second messenger. The presence or absence of the active signal-transducing component is, given the chemical inertness of the ion, dependent neither on an interplay of synthesis and turnover nor on covalent modifications. Instead, globally or spatially restricted elevations in the free intracellular Ca^2+^ level depend on the entry of ions from the cells’ external pool upon the opening of Ca^2+^ ion channels within the plasma membrane (PM) or on the release from internal stores, primarily from the endoplasmic reticulum (ER). A mechanism that liaises both processes is store-operated Ca^2+^ entry (SOCE), interconnecting a drop in the level of Ca^2+^ within the ER-reservoir to the stimulation of PM-embedded channels. This mechanism is triggered by the engagement of cell surface receptors with their corresponding ligands and the subsequent generation of soluble inositol triphosphate (IP_3_) by enzymatic cleavage of phosphatidylinositol 4,5-bisphosphate (PIP_2_), a minor phospholipid of the cellular plasma membrane. After capture of the intracellular messenger by IP_3_ receptors that span the ER membrane and their subsequent activation, the exit of Ca^2+^ from the ER lumen is triggered [3,4,5]. The resulting increase in cytosolic Ca^2+^ is rather limited yet sufficient to trigger Ca^2+^ influx from the extracellular space. Importantly, this store-operated entry holds a dual role by allowing (i) for the induction of diverse Ca^2+^-dependent cellular events and (ii) the re-establishment of cellular rest, as it provides a source of ions for the replenishment of internal stores [3].

Among store-operated channels, the calcium-release-activated calcium (CRAC) channel, initially described in cells of the immune system in the late 1980s and early 1990s, is the best characterized. The CRAC channel is distinguished by an extraordinary selectivity for Ca^2+^, a low single-channel conductance of tens of femto-Siemens (fS) and, on the molecular level, by the indispensability of two proteins residing in spatially separate phospholipid bilayers: Stromal Interaction Molecule 1 (STIM1) within the ER-membrane and Orai1 in the plasma membrane. For a recent review, see Lewis et al., 2020 [6].

CRAC Activation Cascade| The activation of CRAC channels follows a diffusion-trap mechanism. Thereby, a drop in ER-luminal Ca^2+^ levels is sensed by the N-terminal domain of STIM1. The STIM1 luminal portion contains a canonical EF-hand domain, a prototypical Ca^2+^ binding structure that sequesters Ca^2+^ by means of electrostatic interactions with acidic residues that are responsible for capturing Ca^2+^ upon rest. Given constant equilibration between protein-bound and free fractions of the ion, store-depletion forces dissociation of Ca^2+^ from resting-state dimeric STIM1 [7,8,9]. This sets a series of conformational changes in motion, accompanied by alterations in the folding state and interaction of luminal STIM1 domains and the transmembrane segment, as well as of the cytosolic protein domains. Once activated by ER store-depletion, STIM1 oligomerizes and translocates to sites within the ER membrane that are close to the PM, separated by distances of about 10–25 nm only [10,11]. While folded towards the ER-membrane upon rest, the activation of STIM1 includes an extension of its C-terminus vital to allow for interactions with cytosolic domains of pore-forming Orai1 proteins. This direct coupling between STIM1 C-terminal CRAC-activating domain/STIM-Orai-activating region (CAD/SOAR) domains and Orai1 culminate in opening of the CRAC channel [12,13,14,15]. Based on X-ray crystallographic and cryo-electron microscopic data on homologous Orai proteins of the fruit fly *Drosophila melanogaster* showing CRAC channels to be formed of six subunits, the human Orai1 protein is also likely to form hexameric complexes to constitute an active CRAC channel [16,17,18]. Apart from hexameric assemblies forming CRAC channels, Orai1 proteins function as subunits in other channels as well. There, they either function in a store-operated and STIM-regulated manner if associated with members of the canonical type of transient receptor potential proteins (TRPC) or, upon forming pentameric assemblies with the Orai3 isoform, give rise to arachidonate-regulated Ca^2+^ (ARC) channels. The latter are functionally detached from internal Ca^2+^ stores and modulated by a fraction of STIM1 proteins resident in the plasma membrane rather than the ER [19]. Although the series of events that culminate in CRAC channel opening is rather well established, inconclusiveness still must be clarified considering stoichiometric relations of STIM1 and Orai1, conformational transitions within the channel complex leading to the establishment of the conductive state, as well as molecular events of fast and slow Ca^2+^-dependent inactivation. Moreover, a broad spectrum of proteins is believed to support the function of this signalling cascade. In particular, in a physiological context and endogenous levels of protein expression, the literature indicates that CRAC channel function relies on a reservoir of positive and negative modulators for authentic CRAC currents to arise. Exemplifying the presumptive multitude of regulatory proteins associated with the CRAC channel in a direct manner, data of Várnai et al. indicated that Orai1 channels form a macromolecular complex protruding 11-14 nm into the cell interior [20,21]. Analogously, HeLa cells stably transfected with STIM1 and Orai1 led to the detection of Orai1 in extended complexes upon rest (700 kDa), while STIM1 seems to engage lesser interactions in the quiescent state (~200 kDa) but is captured in a complex with also Orai1 of 670 kDa upon store depletion—an observed phenomenon that points to the presence of auxiliary partners within this signalling cascade as well [21]. Interaction partners are eventually directly involved in any step of the activation cascade or serve to establish signalling hubs critical for downstream responses. Furthermore, proteinaceous modulators of SOCE functioning in an indirect manner have been reported, for instance, by creating a distinct lipid microenvironment at ER-PM junctions [22]. Given that interacting proteins and indirect regulators hold vital roles in CRAC channel function but are often left in disregard in the rather STIM1/Orai1-centered research field, the following review focuses on a compacted recapitulation of so far published modulators of STIM1 and/or Orai1.

## 2. Regulators of CRAC Channel Function

### 2.1. Protein Trafficking and Dynamics

Ca^2+^ current amplitude depends on the absolute amount of channel proteins present in the membrane. Consistently, modulation of protein expression and targeting to the respective membrane delineates a primordial regulatory layer of CRAC channel function. In this regard, Orai1 proteins are dynamically internalized from the plasma membrane, whereby the proportion of Orai1 proteins present on the cell surface under resting state and physiological conditions was reported to approximate 40%, while coupling of STIM1 interferes with internalization. In concert with the exocytotic machinery, this shifts the equilibrium towards a preferential plasma membrane residence (~65%) [23,24,25]. The importance thereof is further highlighted given that defective channel trafficking has been shown to lead to serious clinical phenotypes [26,27,28,29,30]. For instance, patients suffering from atopic dermatitis have recently been identified to show an increase in membrane-resident Orai1, leading to a mismatch in the STIM1-Orai1 stoichiometry that phenotypically culminates in the inhibition of Ca^2+^ entry and gene expression [31]. Proteins that are believed to regulate trafficking and dynamics of STIM1 and Orai1 are discussed in the following and are further summarized in Table 1. Apart from proteins involved in cell surface expression of Orai1, endocytosis, and turnover, ER-sculpturing proteins and these involved in the establishment/maintenance of ER-PM junctions are included as well as others regulated by STIM1/Orai1 or those involved in downstream responses, as will be treated in upcoming sections.

Chaperonin-containing T-complex protein 1| Recycling of Orai1 proteins under resting-state conditions is dependent on Rho proteins, as well as Rab5 and chaperonin-containing T-complex protein 1 (CCT, also referred to as TRiC for T-complex protein 1 Ring Complex). Thereby, CCT is considered as the main regulator of Orai1 endocytosis in a manner relying on direct interactions of the concerning protein with a CCT-binding site within the intracellular loop of Orai1 subunits (amino acids 157–167). Explicitly, this interaction seems to promote internalization, given that inhibited CCT-binding was reported to foster cell surface expression upon rest, imposing consequences on SOCE activation, duration, as well as on downstream responses [23,30].

SNAP-25| In regard to protein targeting, the plasma membrane-associated SNARE protein synaptosome associated protein 25 (SNAP-25) was shown to be intricate for activation-related and Ca^2+^-dependent plasma membrane accumulation of Orai1 [25].

SPCA2| Apart from proteins mastering generic reshuffling of Orai1, several mechanisms of Orai1 internalization or plasma membrane enrichment seem to diverge in a cell-type-specific manner. In that context, the secretory pathway Ca^2+^-ATPase SPCA2 was reported to interact with Orai1 to accomplish its forward trafficking in mammary epithelial tissues [32]. Physiologically, the interplay between SPCA2 and Orai1 is important for lactation. However, instead of constituting an actual regulator of SOCE, the interaction with SPCA2 allows for constitutive channel activation irrespective of STIM1, thus accomplishing store independent Ca^2+^ entry (SICE) instead. However, SPCA2 shall be highlighted herein for its clinical relevance. In this regard, Orai1 and SPCA2 were reported to be expressed at aberrant levels in some breast cancer tissues with overexpression patterns correlating with cancer aggressiveness [32,33,34,35].

Findings of Fenech et al. suggest that SPC2C, a C-terminally shortened version of SPCA2 expressed in cells of pancreatic origin, affects store-independent, as well as store-dependent, Ca^2+^ influx. Although co-immunoprecipitation analysis indicated interactions between Orai1 and SPCA2C that correlated with the patterns of STIM1 expression, its modulation of SOCE was intriguingly reported to be independent of this association and to occur in an Orai1-independent manner [36]. In addition, recent experimental data revealed STIM1-independent activation of Orai1 by interactions with the ubiquitously expressed SPCA1a isoform. This association apparently owns clinical relevance as well, considering that impairments thereof relate to Hailey–Hailey disease, a disorder of the skin [55].

Contrasting SPCA2-dependent incorporation into the plasma membrane, CRAC channels are specifically withdrawn from the cell surface in the course of meiosis to hinder store-operated currents from activating. This is accomplished by means of an endocytotic pathway orchestrated by caveolin (Cav), dynamin, and Rab5 and is dependent on a consensus caveolin-binding locus (amino acids 52–60) present in the intracellular N-terminus of Orai1 [23,56]. Apart from meiosis, specific internalization of Orai1 is also observable in epithelial cells of the proximal tubulus. Recent data indicate that the protein amnionless, a critical element of receptor-mediated endocytosis, associates with STIM1-complexed Orai1 after store depletion, allowing for clathrin-mediated, yet caveolin-independent, uptake in a manner correlating with the Ca^2+^-dependent re-uptake of albumin from primary urine. Consequently, downregulation of this process due to changes in the expression level of Orai1 and elevations in the level of steady-state internalization were implicated to underlie progressive losses of normal kidney function in diabetic patients [37].

RHBDL2| Recently, Grieve et al. identified Orai1 as a substrate for the rhomboid-like protein 2 (RHBDL2), a membrane protein that belongs to the family of serine proteases. The authors propose that RHBDL2 regulates Ca^2+^ signalling by optimizing the stoichiometry between Orai1 and STIM1. In this study, the protein was supposed to prevent an inappropriate activation of Orai1 in resting cells, based on a mechanism of conformational surveillance. According to the study, a loss of RHBDL2 led to elevated levels of Orai1, causing a disbalance in Ca^2+^ homeostasis [38].

Calnexin, Exportin1, and Transportin1| The presence of STIM1 within the ER-membrane is vital for its function as Ca^2+^ sensor and SOCE activation. However, a fraction thereof is expressed within the plasma membrane under steady-state conditions, hypothesized to comprise a regulatory effect on Ca^2+^ currents conducted by store-operated channels [57]. Apart from discussions on glycosylation patterns as a decisive factor for cell-surface or ER-membrane expression, a study by Keil and co-workers on hippocampal neurons concerns the ubiquitin-proteasome system exerting a regulatory function on the presence of STIM1 on the cell surface and SOCE. Indeed, exposure of cells to inhibitors of the proteasome elevated plasma membrane expression of STIM1 as well as peak Ca^2+^ currents upon store depletion to a significant extent. In contrast, overexpression of the E3 ligase POSH reduced plasma membrane levels of STIM1 [58]. In addition, Saitoh et al. found that STIM1 variants missing their C-terminal coiled-coil domains are extensively targeted to the plasma membrane, eventually being an indication for a critical role of the concerning segments in ER retention. The same study identified glycosylation-independent binding between calnexin and STIM1, in addition to associations between the latter and a pair of proteins functioning in protein transport, exportin1 and transportin1 [39].

EB1 and +TIP| Other auxiliary proteins relevant to CRAC channel function affect the dynamics of STIM1/Orai1 within the ER- or plasma membrane rather than upstream trafficking. In this regard, STIM1 dynamics are highly dependent on microtubules, cytoskeletal components that are inherently essential for cell viability. The microtubule plus-end tracking protein (+TIP) end-binding 1 (EB1) associates with the STIM1_642-645_ segment, the latter of which belongs to the class of +TIPs as well [59]. In addition to tracking emerging microtubules and allowing for continuous movement of STIM1 throughout the ER membrane if stores are replete, EB1 is involved in ER remodelling. The interaction with STIM1 is hindered upon activation, based on the phosphorylation of a triad of serine residues (575, 608, and 621) that are located in the vicinity of the EB1 association motif, as is accomplished by extracellular signal-regulated kinase 1/2 (ERK1/2) [40]. Thereby, a study indicated that the release of EB-1 is followed by binding of the microtubule stabilizer adenomatous polyposis coli (APC). The latter, inherently also representing an interaction partner of EB-1 and a potent tumour suppressor with genetic alterations correlating with colon cancer, associates with a rather distal STIM1 domain, including the amino acids from position 650 to 685 [41,42]. Knock-down of APC was shown to shift foci of STIM1 clustering from ER-PM contact sites towards regions buried deep within the cell interior with the consequence of impeded coupling to and activation of Orai1. Moreover, experimental findings indicate that the activation-related relay between interactions of both EB-1 and APC with STIM1 is reverted upon deactivation by store-replenishment, implying the dissociation of APC to facilitate re-binding of EB-1 [20,41].

**Figure 1:**
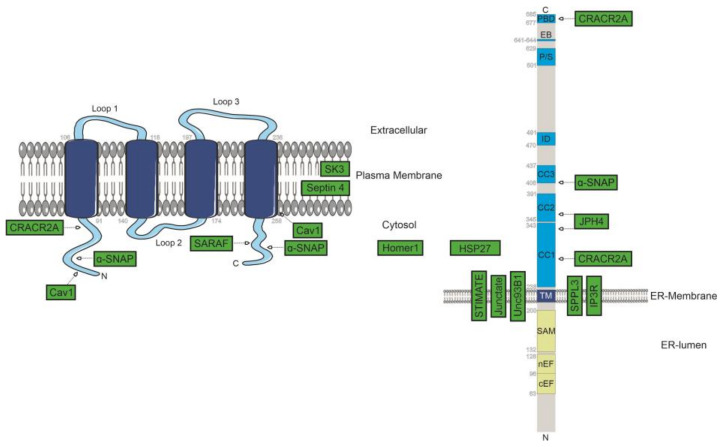
Identified positive regulators of SOCE. Known protein-localisation together with potential identified interaction sites between STIM1/Orai1 and regulatory-interacting proteins are represented in green boxes.

Heat shock protein: HSP27| Heat shock protein 27 (HSP27) is a chaperone that interacts with various proteins. Huang et al. showed that knock-down of HSP27 leads to a reduction of Ca^2+^ influx via a decrease of STIM1. The authors propose that the reduction of STIM1 might be due to a loss of stability of the proteins and not due to transcription. Immunoprecipitation assays indicate that HSP27 interacts with STIM1, but no interaction was observed for Orai1 (Figure 1) [60].

UBQLN1| Lee et al. discovered an interaction between Ubiquilin 1 (UBQLN1), a protein that degrades presenilin and polyglutamine proteins, and Orai1. Ubiquilin 1 leads to an increase of Orai1 ubiquitination and downregulation of channel activation. The authors suggest that Ubiquilin 1 downregulates intracellular Ca^2+^ mobilization by promoting the ubiquitination and lysosomal degradation of Orai1. According to the study, the UBL and UBA domains of Ubiquilin 1 seem to be non-relevant for the interaction with Orai1 [43]. Moon et al. recently published a study indicating that upon ubiquitination, Orai1 is degraded in a Crbn-dependent manner by the proteinase cereblon [61].

### 2.2. ER Sculpturing Proteins, ER-PM Tethers, and PM Microdomains

Reticulon 4| Proteins involved in the regulation of the CRAC signalling cascade yet devoid of physical interactions with both STIM1 and Orai1 eventually possess a critical role in the establishment of a proper ER morphology. Among them ranks the ubiquitously expressed reticulon 4 (RTN4), retained in the ER on the basis of structural elements that map to its C-terminal segment, namely the reticulon homology domain (RHD). This domain comprises two hydrophobic stretches that are intercalated by a hydrophilic segment and are thought to insert into the ER membrane as hairpins. Given that this integration distorts the lipidic organization of the outer leaflet to a higher extent compared to the inner layer, RTN proteins are, in addition to oligomerization in an arc-shaped fashion, critical for the establishment/stabilization of membrane domains of high curvature, such as ER tubules. Consistently, murine embryonic fibroblasts devoid of RTN4 expression exhibited tubulation deficiencies in favour of an enrichment in the appearance of ER sheet structures, while a normal ER function was nevertheless maintained in many respects. However, exposure to thapsigargin (TG), an inhibitor of the sarco/endoplasmic reticulum Ca^2+^ATPase (SERCA), was associated with a lesser magnitude of Ca^2+^ mobilization, in addition to a significant reduction in the consequent Ca^2+^ entry from the extracellular space. This is mechanistically in line with an unavoidable shift in the presence of STIM1 from a normally tubular distribution to sheets due to the overall changes in ER morphology. This likely poses constraints on the diffusion-limited ability of STIM1 to reach ER–PM junctions upon activation [44].

Rather than ensuring just close intermembrane contact to allow for physical STIM1–Orai1 interactions, proteins contributing to the establishment and maintenance of ER-PM junctions hold, eventually in conjunction with modulators of local lipidic compositions, a multifarious regulatory potential on SOCE. While referring to a comprehensive review of Cao et al. [62] or Zaman and colleagues [63] on this matter, a limited set of structural components of ER-PM junctions shall be explicitly mentioned herein, including junctate and junctophilins [45].

Junctate and Junctophilins| Knock-down of junctophilin 1 (JPH1) that bridges the gap intercalated between transverse tubular invaginations of the plasma membrane of skeletal muscle and the sarcoplasmic reticulum (SR), or analogous depletion of junctophilin 2 (JPH2), the latter of which is ubiquitously expressed in any muscle type, was reported to interfere with SOCE and SR Ca^2+^ storage capacity even before the fundamental molecular players of CRAC currents had been unravelled (Table 1) [45,46]. Later, JPH1 was reported to assemble in puncta during store depletion, coinciding with these formed by STIM1, whereas specific JPH2 mutants have been identified to lead to hypertrophic phenotypes of skeletal myotubes in an Orai1-dependent manner [46,64]. Two further types of junctophillins, JPH3 and JPH4, have also been identified, sharing their fundamental architecture with the former variants, including the appearance of phospholipid-binding domains at the N-terminal end and a single transmembrane domain defining the C-terminus to traverse the phospholipid bilayer of the ER. However, these are mainly expressed in cells of the nervous system, while junctophilin 4 was recently also identified to scaffold ER–PM junctions in T lymphocytes. Nevertheless, JPH4 seems to be, at least in the latter cell type, dispensable for the establishment and/or maintenance of ER–PM junctions, since deficiency in this junctophilin was accompanied by neither numeric nor structural changes in the concerning intermembrane contacts. Knock-down of JPH4 was shown to compromise resting-state ER Ca^2+^ content and SOCE in primary T cells and Jurkat T lymphocytes, interfering with effector functions. Consistent with findings that the latter are rescuable by overexpression of STIM1 together with observations that knock-down of the peculiar junctional component truncates STIM1-clusters emerging successively to store depletion in size and number, JPH4 was suggested to be vital for STIM1 relocation upon activation. This is eventually dependent on direct couplings mapping to the first and second coiled-coil domain of STIM1 (CC1 and CC2) and JPH4, the latter of which seems to be constitutively clustered at ER–PM junctions. Indeed, pronounced overexpression of the junctional protein is, according to data of Woo et al. [65], capable of forcing STIM1 to accumulate at junctions even if ER luminal Ca^2+^ reservoirs are replete, yet urging the sensor protein in a conformation unable to activate CRAC channels (Figure 1). Moreover, STIM1 variants deprived of their polybasic PIP2-binding domain, necessary to trap the sensor at ER-PM junctions by means of electrostatic interactions, were shown to be capable of protein-interaction guided clustering at ER–PM contact sites in the presence of junctophilin-4 or junctate. Junctate represents an additional ER membrane-spanning protein and is a putative component of ER-PM contact sites, also in cells devoid of endogenous Orai1 [65,66]. Taking all this into account, it seems plausible that junctional proteins contributing to the recruitment of STIM1 are critical for SOCE under physiological conditions with expression levels of functional Orai1 and STIM1 serving as limiting factors [66]. Direct coupling between junctate and STIM1 is accomplished by ER-luminal sections of both, rather than transmembrane and/or cytosolic segments (Figure 1) [65,66]. 

Interestingly, not only STIM1, but also the 33 kDa measuring junctate, originally described in electrically excitable cells and later also in components of the immune system, is equipped with an EF-hand domain, located at the C-terminus of the protein, able to sense ER-luminal Ca^2+^. In this regard, initial reports of Srikanth and colleagues linking junctate to CRAC channel activation revealed that mutation of Ca^2+^ binding domains in junctate leads to cluster formation of STIM1 under store-replete conditions [66]. Contrasting data on JPH4 overexpression, store-independently clustered STIM1 proteins were indeed reported to be able to induce gating of Orai1 channels [65,66]. In addition, junctate, depletion of which was shown to significantly compromise store-operated Ca^2+^ influx in Jurkat T lymphocytes, seems to comprise a moderate affinity for Orai1 in complex with STIM1, with interactions being promoted successively to ER Ca^2+^ store emptying [66]. Apart from direct associations with STIM1 and/or Orai1, the discussed junctional constituents seem to engage in a complex to synergistically promote SOCE whereby the cytosolic domains of both proteins are apparently critical [65,66]. Such an interplay with other junctional and/or plasma-membrane-embedded proteins is essential for junctate to reside at ER–PM contact sites since it shows a rather uniform distribution throughout the ER membrane upon sole expression [66]. Apart from its role in bridging ER and PM, junctate is relevant for the establishment of ER–phagosome contacts. Spatial increases in intracellular Ca^2+^ levels that are achieved by the STIM1-guided opening of Ca^2+^ channels of the phagosomal membrane boost phagocytic efficacy. In this regard, junctate was shown to be able to recover Ca^2+^ transients and phagocytosis in cells with knock-out of STIM1 or STIM2, respectively [67].

Caveolin-1| The clinically highly relevant Caveolin-1 (Cav1), already mentioned in a previous Section 2.1 in the context of meiotic cell-surface clearance of Orai1, serves, as indicated in Figure 1, as a positive regulator of SOCE in a series of cell types, given that SOCE-specific currents were repeatedly shown to be attenuated upon treatment with Cav1-specific siRNA (Figure 1) [68,69]. However, the molecular-scale function of Cav1 in SOCE is controversial in some aspects. On the one hand, a recent study by Bóhorquez-Hernández et al. [70] identified SOCE activation going along with an association of Orai1 and Cav1. Here, the latter protein that inserts into the phospholipid bilayer in the form of a hairpin was proposed to comprise an affinity to a segment of the fourth transmembrane helix of Orai1, as well as the cytosolic N-terminus, explicitly residues intercalated between the positions 250-258 and those from position 52 to 60, respectively [30,70,71]. Thereby, Cav1 expression might be vital to allow for further important regulatory mechanisms to come into play, namely those imposed by lipids acting upon the accumulation of Orai1 within dynamically assembled subregions of the membrane that are defined by peculiar lipidic compositions [71,72]. This is explained by Cav1 constituting a major scaffolding element of specialized microdomains of the plasma membrane characterized by heightened levels of cholesterol and sphingolipids that form invaginations, termed caveolae [68,69,73,74]. Consistently, overexpression of Cav1 was shown to correlate with elevations in store-operated currents in a manner paralleled by alterations in caveolae count in Hs578/T breast cancer cells, while knock-down lowered the number of these microstructures and silenced SOCE activity to a considerable extent [69]. In addition to its effect on channel function itself, Cav1 plays a direct role in the induction of downstream responses. It serves as an interaction platform for a series of signal-transducing proteins to allow, in concert with the specific lipidic microenvironment, for the establishment of signalling hubs [71,72]. (For review, see Pani et al., 2009 [73].) Thereby, structurally divergent domains of the scaffolding protein have recently been suggested to be vital for the ability of entrant Ca^2+^ to allow for the activation of specific transcription factors [70]. Contradictory to these functions, knock-down of Cav1 in smooth muscle cells of the respiratory system was reported to vanquish SOCE in a manner related to decreases in whole-cell and calveolar Orai1 expression. Conversely, overexpression of Cav1 in the same cell type culminated in elevated levels of Orai1 to be synthesized, in turn promoting Ca^2+^ entry upon store depletion. The same report by Sathish and co-workers hypothesized that the regulatory potential of Cav1 on SOCE was independent of STIM1, given that exposure to Cav1-targeted siRNA left STIM1 levels unaffected, while overexpression of the Ca^2+^ sensor increased SOCE in a manner only partially reversible upon knocking down Cav1 [74]. However, it should be emphasized that a potential effect of Cav1 on CRAC channel expression was not reproducible in other cell types, such as HEK293, wherein neither STIM1 nor Orai1 levels diverged in the presence or absence of Cav1, as found by another group. The promotion of SOCE by Cav1 was alternatively proposed to be due to an enhanced stability of STIM1-Orai1 complexes after store depletion [70].

Septins| Several subtypes of septin proteins serve, as initially revealed in RNAi-based efforts of Sharma et al., as regulators of SOCE in a manner mechanistically related to the initially stated function of Cav1. Following store depletion, septins dynamically reposition from a widespread distribution throughout the plasma membrane to form local accumulations in a manner temporally correlated with STIM1 redistribution and co-clustering of both fundamental elements of the CRAC channel complex. Notably, the foci enriched in septins are spatially separated from STIM1/Orai1 clusters and in relation to the specific septin subform, impose different functional consequences on SOCE yet accomplished in an indirect manner [75,76]. For a detailed review of the role of septins in SOCE, see Deb et al., 2016 [77]. The septins SEPT2, SEPT4 and SEPT5, collectively constituting filament-forming GTPases, were, according to analysis in Jurkat T lymphocytes and HeLa cells, implicated as positive modulators of SOCE (Figure 1). This is mastered by a septin-dependent enrichment of PIP2 and eventually PIP3 at sites of close ER-PM contact that promotes, based on electrostatic interactions between the polybasic domain of STIM1 and the charged phospholipid head groups, arrest of extended-state STIM1 and in turn, STIM1-dependent recruitment of Orai1 to ensure efficient Ca^2+^ influx [75]. This is consistent with findings of other groups, including reports that depletion of SEPT4/5 correlates with a downturn in the density of STIM1 clusters [78]. Apart from the reshuffling of PIP2 during activation, septins regulate the distribution of membrane lipids in the resting state, accounting for observations that the ablation of specific forms correlates with alterations in Orai1 distribution upon rest [75].

**Figure 2:**
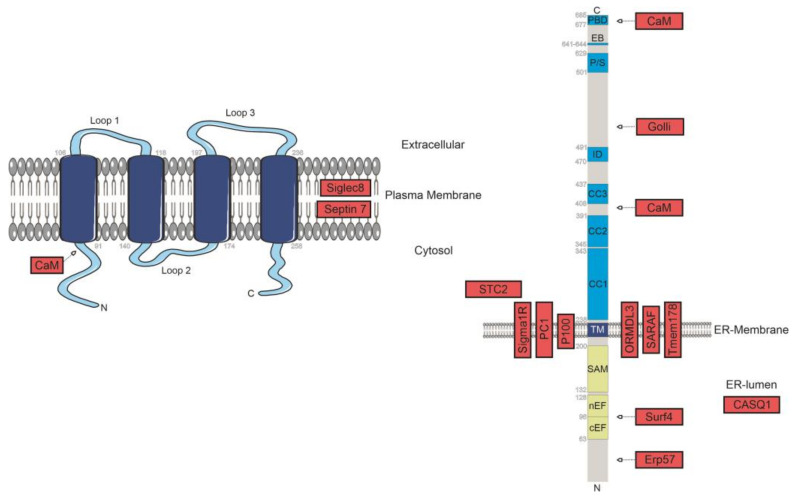
Identified negative regulators of store-operated Ca^2+^ entry (SOCE). Known protein-localisation together with potential identified interaction sites between STIM1/Orai1 and regulatory-interacting proteins are represented in red boxes.

Moreover, Katz and co-workers proposed that SEPT4/5 indirectly modulate the number of ER–PM contact sites and slow the diffusion of Orai1 detaching from STIM1. Here, TG-treatment was reported to locally confine motions of Orai1 that were in parts negotiated by knock-down of SEPT4/5 and restorable by SEPT4 expression, whereas diffusion characteristics of STIM1 upon rest or after store depletion were insensitive to changes in SEPT4/5 expression. The lower mobility in the vicinity of ER-PM junctions would in turn foster rebinding of CRAC channels based on elevations in their local concentration, whereas initial recruitment of Orai1 to the junctions was stated to be exclusively accomplished in a STIM1-dependent manner but irrespective of septins [78]. Apart from the former septin proteins, a modulatory role of SEPT7 on Ca^2+^ entry has been established yet is divergent in several respects [76,79]. On the one hand, SEPT7 was shown to downregulate store-operated currents if overexpressed, whereas on the other, lessening of its levels allows for store-independent Ca^2+^ entry in *Drosophila* neurons and elevations in basal cytosolic Ca^2+^ levels (Figure 2). This is putatively explained by alterations in the structure of residual septin filaments that serve as mimetic of another store-dependent remodelling that is indispensable for PIP2-reorganizations [76]. Furthermore, a recent study identified the human SEPT7 as a critical regulator of Orai1-driven Ca^2+^ entry into neuronal progenitor cells and differentiated neurons. Although the underlying mechanism remains to be elucidated, it seems to deviate from the one declared for the homolog of fruit-fly, given that the polybasic segment of the protein was shown to be indispensable for human SEPT7 to exert its regulatory function rather than the GTPase domain as relevant in the case of the former [79].

### 2.3. Positive Modulators of SOCE

Apart from the previously treated proteins that regulate CRAC channel activity in a rather indirect manner, for instance by modulating cell surface expression of Orai1, protein turnover or membrane structuration, others directly assist in CRAC channel activation at different stages of the multilayered activation cascade. These positive modulators, for which a graphical summary that further includes indirect regulators is provided in Figure 1, are discussed in the following section.

α-SNAP| By knock-down analysis in *Drosophila* Kc cells, Jurkat T lymphocytes, HEK293 cells or U2OS cells, Miao and co-workers revealed a thereto unseen, direct role of soluble N-ethylmaleimide sensitive fusion protein (NSF) attachment protein (SNAP) of fruit-fly and the ubiquitously present mammalian α-SNAP in the onset of store-operated Ca^2+^ influx that was in the case of the latter protein restorable by overexpressing the closely related β-SNAP, expression of which is physiologically limited to the brain. Despite the inherent function of SNAP in vesicle fusion, depletion of α-SNAP renders plasma-membrane expression and ER-membrane localization of Orai1 and STIM1 untouched. Instead, knock-down of α-SNAP was identified to lead to increases in the dimensions of STIM1-Orai1 clusters with normal STIM1 densities but unproportional elevations in Orai1 count. Consistently, overexpression of the modulator was reported to augment Ca^2+^ influx in correlation with higher per-cluster densities of STIM1. Thereby, the regulatory potential of α-SNAP seems to rely on direct protein–protein interactions, involving CAD of STIM1, as well as both cytosolic tails of Orai1, yet in view of co-immunoprecipitation and pull-down efforts, comparatively weak in the case of the N-terminus (Figure 1). Despite a certain level of constitutive association, the proportion of α-SNAP bound to STIM1 elevates in correlation with the emptying of ER Ca^2+^ reservoirs, whereas lacking, in the absence of STIM1, co-localization with Orai1 irrespective of the filing state of the ER. Nonetheless, under conditions of co-expression of STIM1 and Orai1, α-SNAP was observed to preferentially co-localize with clusters containing both STIM1 and Orai1. Taken together, these data seem to, with regard to α-SNAP, have functional significance for the establishment of appropriate STIM1/Orai1 ratios co-agglomerating upon store depletion [80].

However, in a subsequent study by the same group, α-SNAP was proposed to function in the association of dimeric Orai1 into multimeric, Ca^2+^-selective channels. Thereby, Li et al. reported that Orai1 formed dimers in mouse embryonic fibroblasts under resting cell conditions, while dynamically engaging assemblies of dimeric, tetrameric or hexameric stoichiometry following store depletion. Ablation of α-SNAP was therein found to lead to a shift towards hexameric Orai1 associations and, concomitantly, to a downturn in the proportion of dimeric and tetrameric complexes. The former assemblies were reported to be capable of conducting sodium currents to considerable extents in a regime insensitive to increases in the level of STIM1 interaction [81]. Indeed, further elucidation of the role of α-SNAP in STIM1/Orai1 function recently demonstrated shortcomings in CD4 T cell signal transduction due to reduced α-SNAP expression mechanistically related to sodium currents conducted by Orai1 rather than SOCE [82]. Moreover, concatemers of Orai1 with a pair of SOAR segments appended to the C-terminus, otherwise conducting constitutive Ca^2+^ currents, were observed to significantly lose Ca^2+^ influx in favour of sodium entry in cells hampered in α-SNAP expression. Seemingly contradicting this is that if expressed as soluble fragments, CAD/SOAR was reported to be competent of re-establishing Ca^2+^ influx also in cells with compromised α-SNAP expression. In any case, STIM1–STIM1 interactions were, in the study of Li et al., devoid of any regulatory potential imposed by α-SNAP, and similar to the preliminary report, depletion of the modulator was per se found to allow for STIM1-Orai1 co-association, lacking apparent influences on STIM1 coupling to the C-terminal section of Orai1. Instead, cells knocked-down in α-SNAP expression were significantly compromised in interactions between STIM1 and the N-terminal Orai1 domain, hypothesized to account for the observed higher mobility of Orai1 in the region of ER–PM junctions. These data might indicate that α-SNAP is required for conformational transitions of the Orai1 N-terminus indispensable for efficient CRAC channel activation [81].

CRACR2A| A regulatory protein that has affinity to STIM1 and Orai1 is CRAC regulator 2A (CRACR2A also referred to as EFCAB4B or FLJ33805) originally discovered by Srikanth et al. According to bioinformatic analyses, CRACR2A, which predominantly locates to the cytosol, is equipped with a pair of EF-hand domains in the N-terminal region and contains, in addition to a leucine-rich element, a coiled-coil domain in its C-terminal section. The homolog CRAC2B that was identified at the same time shows conserved residue identities within the putative EF-hand motif and the C-terminal coiled-coil domain and shares a sequence identity of 36% with human CRACR2A. The interactions among CRAC2A and STIM1 or Orai1 involve the coiled-coil domain, as well as the proline/lysine rich segment of the former, and considering Orai1, map to the N-terminal domain with K85 and K87 serving as critical interaction sites (Figure 1) [21]. It is worth mentioning that the sites of CRACR2A-STIM1 and CRACR2A-Orai1 interaction overlap with these described to bind calmodulin (CaM) in the presence of Ca^2+^, explicitly including hOrai1_68-91_ and the lysin-rich domain of hSTIM1 [21,83]. CRAC2A associations with both basic components of the CRAC channel complex are associated with a stringent dependence on the activation state, as these are promoted successively to store depletion yet exclusively under Ca^2+^-free conditions, while otherwise strong binding between the mentioned domains of STIM1/Orai1 and CRACR2A was reported to vanquish if exposed to buffer solutions supplemented with 2 mM Ca^2+^ [21]. In view of its EF-hand domain, CRACR2A was supposed to serve as a sensor of cytosolic Ca^2+^ levels, dissociating from the CRAC channel complex if concentrations of the ion are elevated. Consistently, an EF-hand mutant devoid of Ca^2+^ binding was curtailed from Ca^2+^ sensitive binding as well. Supplementing in vitro analysis, total internal reflection fluorescence (TIRF) microscopic analysis of fluorescently labelled STIM1 proteins or co-transfection of STIM1-mCherry and Orai1-GFP showed that store-depletion dependent clustering of STIM1 at ER-PM junctions is significantly reduced in CRACR2A-silenced Jurkat T cells compared to controls. Similarly, Orai1 clustering is strictly dependent on the presence of CRACR2A, while hindered by its depletion. Conversely, expression of the Ca^2+^-insensitive CRACR2A form, together with STIM1-YFP, led to variable clustering already under resting state conditions, while resulting in extended clusters after store depletion, and was in the longer term shown to considerably elevate the proportion of Jurkat T cells undergoing apoptotic cell death compared to these expressing solely endogenous CRACR2A or counterparts transfected with wildtype CRACR2A. Further, expression of CRACR2A in HeLa cells stably expressing STIM1 and Orai1 was associated with a significant increase in the maximum of SOCE, while EF-hand mutants not only led to elevations in peak [Ca^2+^]_int_, but also in persistency. Altogether, CRACR2A likely stabilizes the association of Orai1 and STIM1 upon store depletion as long as intracellular Ca^2+^ concentrations are low. However, further data are necessary to decipher whether the cytosolic EF-hand protein is actively involved in the accumulation of STIM1 and/or Orai1 at ER-PM junctions or recruited to already clustered proteins in a subsequent step. Although CRACR2A was reported to be more potent in enhancing Orai1-dependent Ca^2+^ influx, CRAC2B seems to show a functional redundancy with the former [19,21]. However, both proteins seem to differ in view of tissue-specific expression levels, with CRACR2A constituting the prevailing form in thymus and spleen, as well as the Jurkat T cell line, while CRAC2B is in greater abundance in HEK293 cells. Consistently, knock-down of the expression of the 2B homolog showed solely mild effects on SOCE in Jurkat T cells compared to CRACR2A knock-down, while outweighing the impacts on store-dependent Ca^2+^ entry in HEK293 cells [21]. In addition to the aforementioned cytosolic form of CRACR2A (CRACR2A-c), a longer variant, CRACR2A-a, has been identified. However, this isoform that is present in vesicles is equipped with a Rab GTPase domain at the C-terminal end unseen in the shorter CRAC2A protein, accounting for functional differences and shall thus remain neglected herein [84]. For review, see Srikanth et al., 2017 [85].

STIMATE| Through genome-wide RNAi screens to identify modulators of cellular Ca^2+^ signalling, Quintana et al. found a protein in the ER membrane encoded by the *TMEM110* gene (Figure 1), which regulates the long-term maintenance and short-term remodeling of ER–PM junctions [86]. This protein, later named STIMATE (STIM-activating enhancer), acts as a positive regulator of SOCE in vertebrates and works in concert with other proteins in the dynamic remodelling of the junctions during signalling (Figure 1). STIMATE has been shown to directly interact with STIM1 to promote STIM1 conformational switch, and depletion of STIMATE reduces STIM1 puncta formation and suppresses Ca^2+^ dependent NFAT signalling [87,88].

Homer| An additional protein that has a positive regulatory effect on SOCE is Homer 1a, as initially described in a study of Jardin-Polo et al. on platelet function (Table 1) [47]. In general, Homer proteins, encoded by three different genes whereof each gives rise to a set of transcripts, serve as scaffolds, capturing proteins intermingled in Ca^2+^-dependent signal transduction and/or regulate these in an isoform-dependent fashion [89]. Homer family members are collectively defined by an Ena/VASP homology 1 (EVH1) region within the N-terminal domain, allowing for the binding to proline-rich segments (PPXXFR) of interaction partners, including IP3R and TRPC proteins [90,91]. Among the proteins derived from the Homer 1 gene, Homer 1a is smallest with the EVH1 domain of Homer 1a being only extended by a short C-terminal segment. Instead, elongated C-termini of alternative gene products comprise a coiled-coil domain that allows for concomitant interactions with equivalent domains of other Homer proteins and capture of targets to be regulated. The shorter, only monomeric variant was in turn implicated as a negative regulator of signalling lattices scaffolded by coiled-coil domain-containing forms, since it recognized the same sequence motif within target proteins but lacked interactions with other Homer proteins [89]. Consistently, the C-terminally extended Homer 1c was found to regulate store-operated Ca^2+^ channels with different consequences on function [92,93].

In the aforementioned attempt of Jardin and colleagues, the Homer 1a isoform was shown to interact with STIM1 upon store depletion and in a Ca^2+^ dependent manner, given that chelation of cytosolic Ca^2+^ impeded this complexation. Although Orai1 proteins also co-immunoprecipitated under the same conditions, the group suggested this apparent association with Homer 1 to be indirect and accomplished by concomitant binding of STIM1 (Figure 1). In conjunction with functional data revealing that interference with Homer 1a binding lacked impacts on the activation of Ca^2+^ influx and initial current characteristics, the hypothesis was raised that early steps of STIM1-Orai1 coupling occur independently of Homer 1a. Instead, Ca^2+^-promoted association of the specific regulator was proposed to subsequently ensure that the complex formed by STIM1 and Orai1 is maintained also under conditions of rising intracellular Ca^2+^ levels, given that ablation of Homer 1a association correlated with considerable impairments in Ca^2+^ entry [47]. The regulatory role of Homer 1 on SOCE has recently been implicated as a promising pharmacological tool for the treatment of conditions related to damages of the blood vessel wall, as in the case of diabetic, atherosclerotic, and hypertensive conditions [94]. This is explained by Ca^2+^-dependent dedifferentiation, proliferation, and relocation of vascular smooth muscle cells into the lumen of blood vessels, accounting for the associated pathological phenotypes. Orai1, STIM1, and several members of the TRPC family were shown to be expressed at higher levels upon injury, whereas Homer 1 expression was altogether just detected in injured cells [94,95,96]. In this regard, Jia et al. reported that any of these components involved in store-operated Ca^2+^ entry interact in a direct manner upon injury, including binding of Homer 1 to Orai1 and multiple TRPC-type proteins. Thereby, knock-down of Homer 1 was shown to lead to a similar effect on SOCE in cultured rat aortic vascular smooth muscle cells, manipulated to model injured counterparts, as treatment with siRNA targeting STIM1, while concomitant ablation of the scaffolding protein and the latter revealed an additive inhibitory effect on Ca^2+^ entry [94]. Apart from pathogenic alterations of vasculature, Homer 1a function in SOCE seems to be relevant for injured neurons as well in a manner related to STIM1 and TRPC channels rather than Orai1. There, modulators of SOCE have been suggested as potential targets for the treatment of Parkinson's disease (PD). Treatment of an in vitro model of PD neurons with STIM1-targeted siRNA or pharmacological antagonists of SOCE increased cell viability and lessened the rate of apoptotic cell death, either of which correlated with elevations in the Homer 1a mRNA levels compared to untreated controls. Intriguingly, the positive effects of SOCE inhibition on cell viability were in parts reverted upon ablating Homer 1a expression [97]. Apparently contrasting with the latter, a study on a non-specific inhibitor of SOCE (SKF-96365) is related to a significant downturn in Homer 1 expression levels upon inhibitor application, whereby protective effects of SOCE inhibition on the same PD model system were posed to relate to a regulatory role of Homer in ER Ca^2+^ levels [90]. This seeming contradiction in terms of Homer 1 expression might be explained by the former study explicitly investigating the Homer 1a variant, while a closer specification of the protein variant is lacking in the latter effort. 

SPPL3| The signal peptide peptidase-like 3, an ER-localized intramembrane aspartyl protease, is a member of the signal peptide peptidase (SPP) family, which is known for its activity as a sheddase [98,99]. The latter includes proteases that are specifically responsible for the withdrawal of ectodomains from membrane proteins, a post-translational process that is vital for an appropriate function of many membrane standing proteins and modulation of their expression levels [100]. A study by Makowski et al. proposes SPPL3 as a positive modulator of the SOCE pathway, as NFAT activation was apparently promoted in the presence of SPPL3 (Figure 1). However, this seems to be accomplished by SSPL3 serving as promoter of STIM1-Orai1 associations rather than related to its proteolytic function. Intriguingly, the protein was even found to be indispensable for the activation of Orai1 by constitutively active STIM1 mutants. These functions of SPPL3 were reported to rely on its association with STIM1 at the transmembrane domain and, eventually, the cytosolic CAD, ameliorating interactions between the latter and Orai1. STIM1 as well as cytosolic CAD fragments of STIM1 were affected by SPPL3 knock-down and immunoprecipitation studies showed associations of SPPL3 with STIM1 [99].

SK3| A growing number of experimental data enforce that ion channels composed of Orai1 engage signalling complexes with other types of ion channels, allowing for a reciprocal regulation in terms of activity [101,102,103,104]. A study by Guéguinou et al. identified a complex formation in lipid rafts between the small conductance calcium-activated potassium channel 3 (SK3), also known as KCa2.3, with Orai1 as well as TRPC1 initiated by activated STIM1 (Figure 1). The authors proposed the existence of a positive feedback loop in which SOCE activates the Akt pathway as well as SK3 channel activity, which leads to SOCE amplification [102]. This association of SK3 and Orai1 was identified to be highly relevant for the migratory abilities of cancer cells [105]. Activation of the cAMP-PKA cascade was shown to lessen the activities of sole and Orai1-complexed SK3, hindering Ca^2+^ influx and in turn, mobility of cancer cells [101]. Apart from SK3, the small-conductance Ca^2+^-dependent potassium channel SK4, also referred to as KCa3.1, relies on store-depletion-guided Ca^2+^ influx via Orai1 channels to gate open, as does the large-conductance Ca^2+^-activated potassium channel BKCa, in turn shifting membrane potential towards hyperpolarized values. This ensures the continuation of Ca^2+^ influx and replenishment of the ER stores by maintaining a high driving force of Ca^2+^ influx and eventually regulates myocyte contraction [103,104].

Unc93B1| Unc93B1, located in the ER membrane of several cell types, is composed of 597 amino acids and shows 12 transmembrane domains. Upon cell stimulation, Unc93B1 can re-localize into endosomes or lysosomes and plays an important role in innate and adaptive immunity by regulating Toll-like receptor (TLR) signalling [106,107]. Several studies identified a loss-of-function mutation on position 412 (H412R), which leads to disruption of the interaction between Unc and Toll-like receptors TLR3, -7, and -9 in dendritic cells but also stops antigen-cross presentation independently of TLR help [108]. Next to the regulation of TLR-signalling, Maschalidi et al., as well as Beutler et al., identified Unc93B1 as a possible interaction partner of STIM1 (Figure 1). They postulated Unc93B1 as a further regulator of SOCE and Ca^2+^ homeostasis, STIM1 Ca^2+^ signalling activity together with Unc93B1 function being essential for MHCI antigen cross-presentation [109,110]. Reduced interaction between STIM1 and Unc93B1 H412R mutants was observed in this regard, as well as a potential wildtype Unc93B1 STIM1 EF-hand domain interaction. Maschalidi et al. assume that Unc93B1 acts as chaperone, which helps STIM1 in the early stages of the oligomerization process. Next to the help in oligomerization processes, Unc93B1 could play an important role in the transfer of STIM1 to ER/PM junctions, activating Orai1 proteins, as was shown by TIRF-microscopy experiments. The linkage between Unc93B1 and STIM1 together with Orai1, leading to Ca^2+^ influx from outside the cells and hence resulting in phagosomal Ca^2+^ hotspots, which are needed for phagocytosis, phagosomal maturation, and antigen-cross presentation, is suggested to be a potential target for further investigation but is not known in detail [109].

### 2.4. Negative Modulators of SOCE

In addition to direct binding partners of STIM1/Orai1 fostering the activation or maintenance of the active state of CRAC channels, a set of proteins has been identified that is vital for the prevention of spontaneous activation, as well as for the cessation of Ca^2+^ influx by aiding channel de-or inactivation, respectively. The next paragraphs review findings on negative modulators of CRAC channel function, as are, together with some indirect modulators, illustrated in Figure 2.

SURF 4| Several negative modulators of SOCE mediate their regulatory impact in a STIM1-related manner. Among these is the surfeit locus protein 4 (Surf 4) that exhibits an affinity to the N-terminus of STIM1 and binds in a manner sensitive to site-directed mutagenesis in the EF-hand domain of STIM1 (Figure 2). According to Fujii et al., hindered Surf4 expression correlates with considerable increases in SOCE and notable increases in STIM1 cluster formation at sites of close ER-PM contact, indicating a modulatory function of Surf4 on STIM1 junction targeting and/or clustering [111].

ERp57| The 58 kDa measuring ER-resident thiol oxidoreductase ERp57 exhibits, apart from its function in the proper folding of emerging glycoproteins, a downregulating activity on store-operated Ca^2+^ influx by serving as binding partner of the ER-lumen facing section of STIM1 (Figure 2). ERp57 deficiency was reported to considerably elevate SOCE and allowed for constitutive STIM1 puncta formation, while overexpression of the specific modulator slowed translocation of STIM1 in a significant manner. In the same study, a pair of putatively disulfide-linked cysteines, STIM1 Cys49 and Cys56, has been implicated as a presumptive interaction site for ERp57 and, given that concomitant alterations of both interfered with Ca^2+^ influx as well, as intricate for STIM1 translocation. The inhibitory association of ERp57 might be overcome under conditions of oxidative stress, whereby S-glutathionylation of Cys56 eventually prevents the oxidoreductase from binding to foster on the other hand puncta formation and SOCE [112].

TMEM178| Another component integrated with the process of prevention of excessive CRAC channel activation is TMEM178, suggested to interact with Gly225 in the transmembrane region of STIM1. Binding of TMEM178 seems to be dependent on the activation state of STIM1, given that co-immunoprecipitation experiments revealed that the Ca^2+^-insensitive and hence constitutively active STIM1 D76A variant was markedly relieved of TMEM178 engagement (Figure 2). In a further sense, the interaction between the Ca^2+^ sensor and the regulator likely occurs in concurrence with STIM1-Orai1 coupling, considering that upon subducting STIM1 variants with compromised amino-termini from SOAR to impede capture of Orai1, TMEM178 binding was regenerated. This led Yang et al. to suggest that the association poses constraints on STIM1 translocation and interferes with its assembly in puncta. Indeed, cells depleted of TMEM178 were reported to depict increases in the size, number, and intensity of STIM1-YFP formed puncta upon TG treatment, while ectopic expression of TMEM178 in HEK293T cells affected the establishment of STIM1 clusters in the adverse manner. Considering control cells expressing the regulator, TG-induced store depletion lessened STIM1-TMEM178 interactions but promoted co-clustering of STIM1 and Orai1. Importantly, the regulatory relevance of TMEM178 in SOCE seems to apply for a rather limited spectrum of cell types, so far reported for osteoclasts and bone marrow monocytes/macrophages, whilst apparently absent in T lymphocytes [113]. Notwithstanding the apparent lack in terms of universality, TMEM178 constitutes a SOCE regulator of high clinical significance. This traces back to TMEM178 representing a putative main modulator of Ca^2+^ entry in inflammatory processes with loss of expression fostering the synthesis of inflammatory cytokines by macrophages. Considering TMEM178-null mice, phenotypes show a higher susceptibility for LPS-evoked sepsis and pronounced inflammatory bone loss [113,114].

PC1/P100, PC2 and IP3R| An important regulatory step in CRAC current activation is store depletion, which involves STIM1 proteins, given that those have been identified to directly interact with the Ca^2+^-release channel IP_3_R. This association was further reported to be fostered upon Polycystin-1 (PC1) expression, a protein comprising 11 transmembrane segments shown to hinder IP_3_R from activating [115]. PC1 itself was also reported as capable of associating with the ligand-capturing domain of IP_3_R to interfere with Ca^2+^ release, as well as with STIM1 to impede its translocation upon store depletion (Figure 2) [116,117]. Considering the latter, coexistence of PC1 and several cleavage products thereof have been identified, whereof the 100 kDa measuring P100 fragment that comprises the C-terminal-most six transmembrane segments and consequent stretches shares the abilities of full-length PC1 to inhibit SOCE by hindrance of STIM1 re-location to and clustering at ER–PM junctional sites (Figure 2) [117]. A later report by Santoso et al. indicated the regulatory effect of PC1 on Ca^2+^ release to be dependent on the PI3K/Akt signal transduction cascade since PC1 expression in Mardin Darby canine kidney (MDCK) cells was associated with increases in the levels of Akt phosphorylation. Interference with the concerning pathway was shown to be able to restore ER store depletion in a manner correlating with abolished STIM1-IP_3_R interactions also in the context of PC1 expression. PC1-dependent modulation of the PI3K/Akt system seems to orchestrate interactions of another polycystin with IP_3_R proteins, namely Polycystin-2 (PC2). PC2 is a non-selective, Ca^2+^-permissive cation channel that traverses the membrane of the ER six times and serves as a positive regulator of Ca^2+^ release by prolonging IP_3_R opening upon IP_3_ engagement via direct interactions.

The interaction of PC2 and IP_3_R is in direct concurrence with the engagement of the latter by STIM1, both of which depend on ER-luminal Ca^2+^ but in a reverse manner and imposing functional consequences on store depletion. In turn, the PI3K/Akt cascade was suggested to promote associations of PC1/STIM1 with IP_3_R if active, concomitantly restraining interactions between PC2 and the particular Ca^2+^ release channel to keep the latter quiescent [115]. Notwithstanding, Sampieri et al. found that IP_3_R associates with STIM1 upon Ca^2+^ store depletion. The interaction of IP_3_R with STIM1 results in an enhanced STIM1 puncta formation and larger whole-cell currents, as well as increased Ca^2+^ influx. However, depleting ER stores with TG does not induce IP_3_R-STIM1 association, indicating that the association requires an active IP_3_R. Enhanced calcium depletion when IP_3_R is overexpressed was observed. Mechanistically, the accumulation of activated IP_3_R in the vicinity of STIM1 puncta was conceived to account for disproportionally high levels of Ca^2+^ depletion close to the Ca^2+^-sensing domain of STIM1, further fortifying clustering and CRAC channel activation [118].

Golli| Shortly after the identification of STIM1 as an indispensable component of the CRAC channel and even before Orai1 was unravelled as the second key element of CRAC current activation, golli proteins had been, based on analysis of Feng et al. in 2006, implicated as negative regulators of SOCE. This is explained by Ca^2+^ imaging analysis and electrophysiological recordings revealing that silencing of golli expression in T cells enhances the influx of Ca^2+^ from the extracellular space after store depletion [119]. The class of golli proteins, derived from the usage of the most upstream of three alternative transcription start sites of the myelin basic protein (MBP) gene, are, apart from the N-terminal sequence, related to prototypical MBPs in terms of structure [19,22,120]. Whereas the latter constitute a main structural component of myelin layers and consistently show predominant expression in cells of the nervous system, golli proteins show rather ubiquitous expression patterns. These proteins, whereof the denomination relates to their initial description and detection as gene products expressed in cells of the oligodendrocyte lineage, are particularly abundant in cells and tissues of the immune system but also the brain [22,119]. Considering the former, the alternatively spliced golli-BG21 isoform, prior-ranking investigated in T-cells, is by far the predominant variant [22,120,121]. Here, the expression level of golli was reported to vary in a fashion dictated by the developmental stage of cells of the T lymphocyte lineage [122]. The importance of tight regulation of SOCE by golli throughout T cell development is highlighted by data that naïve CD4^+^ T-cells, synthesizing golli under physiological conditions, showed hyperproliferative phenotypes after stimulation if isolated from golli-deficient mice [119,122]. Consistently, there are reports that IL-2, a critical driver of T cell activation and division, is expressed at higher levels in golli-deficient cells after stimulation compared to controls, whereas earlier data revealed that extraneous expression of golli in Jurkat T lymphocytes interfered with IL-2 promoter-related reporter protein expression [119,120]. The negative effect of golli on CRAC channel activity seems to be in line with its plasma membrane association, achieved by means of an attached myristoyl moiety. This assumption is based on confocal fluorescence microscopic analysis and biochemical evaluations, revealing that mutagenesis on golli to interfere with the particular type of post-translational modification vanquished SOCE inhibition [119]. Consequent investigations of the function of golli proteins on the molecular-scale of Walsh and colleagues identified that they interact with the cytosolic segment of STIM1 (Figure 2). Moreover, interactions between STIM1 subducted from transmembrane and ER-luminal domains and golli, monitored in a BiFC (bimolecular fluorescence complementation) format based on the re-assembly of EYFP, were observable in regions close to the plasma membrane exclusively after store depletion. This is rather surprising, considering that expression of isolated C-terminal domains of STIM1 has been reported to support, due to binding to Orai1, constitutive targeting of the concerning STIM1 fragments to the plasma membrane and thereby culminate in store-independent Ca^2+^ influx. This seems to be explained by a modulatory potential of intracellular Ca^2+^ levels on STIM1- golli-BG21 interactions, given that histamine treatment of HeLa cells co-expressing the concerning BiFC constructs to evoke transient alterations in cytosolic Ca^2+^ concentrations allowed for the re-establishment of EYFP emission as well, in addition to Ca^2+^-dependent binding observed in biochemical efforts [123].

SARAF| A further regulator of SOCE that recently gained marked interest is the ER-membrane standing protein SOCE-associated regulatory factor (SARAF). Initially identified by Palty and colleagues, SARAF has been shown to be critical for slow Ca^2+^-dependent inactivation of CRAC channels [124] (Figure 2). SARAF, later also identified to be eventually expressed in the plasma membrane in a manner dictated by surface-localized STIM1, engages dynamic interactions with STIM1 upon rest and in the course of activation, in addition to store-depletion dependent direct couplings to Orai1 [125,126]. (For review, see Jardín et al. 2018 [127].) Thereby, emptying of the ER-luminal Ca^2+^ reservoir was shown to evoke constitutively bound SARAF to detach from STIM1, concurrently promoting interactions with Orai1, followed by dissociation from the latter in favour of STIM1-rebinding. Although SARAF hinders CRAC channels from activating if overexpressed and promoting SOCE if depleted, the interaction with Orai1 may eventuate Orai1 channel activation in a STIM1-independent manner and was shown to lead to a transient enhancement of Ca^2+^ entry [126]. ER-resident SARAF interacts with STIM1 via its cytosolic domain and translocates to ER-PM junctions upon store depletion in a STIM1-dependent manner [128]. Considering STIM1, the segment intermingled between the residues 448 and 530 that is appended to the SOAR is vital for SARAF interactions. The concerning conserved domain is referred to as C-terminal inhibitory domain or CTID and is constituted of two lobes. The final segment of the CTID, commencing at position 490, is assumed to guide SARAF towards the SOAR domain and in particular to a cluster of lysine (_382_KIKKKR_387_) indispensable for SARAF binding. On the other hand, the STIM1_448-490_ section hinders, based on associations with the STIM1 CC1 residues _318_EEEE_322_, SARAF from approaching its cognate binding site [129]. Interactions between SARAF and STIM1 of the former kind are likely involved in the prevention of spontaneous activation of CRAC entry if stores are replete, while activation of STIM1 eventually enforces the release of SOAR from SARAF-capture to access Orai1 instead. Consequent re-engagement of interactions between SARAF and SOAR finally launches slow inactivation [124,126,129,130]. Although interactions map to the cytosolic domain, the proportion of SARAF that resides within the ER seems to choreograph inactivation by responding to alterations in luminal Ca^2+^ concentrations. The concerning domain has recently been crystallized by Kimberlin and colleagues, indicating a pair of such domains to engage a domain-swapped dimer that seems to be on the functional level critical for inactivation of store-operated currents [128]. Notably, mechanisms of SARAF-determined inactivation seem to exclusively affect CRAC channels resident within plasma membrane subcompartments enriched in PIP_2_ that are believed to be populated just after targeting STIM1-Orai1 CRAC channel complexes transiently to regions of marginal densities of this peculiar phospholipid [126,127,129,130,131].

Recent data by Lopez et al. indicated that STIM1–SARAF interactions relate to tyrosine phosphorylation, given that STIM1 Y316F constructs were revealed to ameliorate STIM-SARAF interactions after store depletion, promoting SCDI in the further sense [132]. The modulation of SOCE by SARAF seems to comprise high clinical relevance, considering recent reports on altered transcript levels of SARAF in patients suffering from pancreatitis, as well as transient interactions between SARAF and STIM1 together with increases in Ca^2+^ entry in cells originating from mice with induced pancreatitis, implicating that restoration and/or stabilization of SARAF might pose promising treatment strategies [133]. In addition, a knock-down in the expression level of SARAF yet concomitant increases in these of Orai1 and STIM1 have recently been shown to correlate with cardiac hypertrophy, the latter of which was attenuated upon overexpressing SARAF in a mouse model system [134]. SARAF was further reported to be, in analogy to STIM1, downregulated following cerebral ischemia and might thereby pose a target of future stroke therapies. Given that neuroprotective attempts alleviated downregulation of SARAF only, preservation of expression was speculated by La Russa et al. to allow for an alternative means of ER replenishment [135]. Another protein that salvages a regulatory role in SOCE, EF-hand domain family member B, EFHB, seems to mediate its function by inducing SARAF and STIM1 to transiently dissociate upon store depletion and in turn, was, in view of RNAi- and overexpression analysis, reported to be relevant for the appearance of STIM1–Orai1 interactions [136].

CaM| Calmodulin (CaM), one of the most prominent calcium-binding proteins, was reported to have an affinity to both STIM1 and Orai1 (Figure 2). CaM is well-established to modulate the activity of a multitude of kinases, phosphates, and transcription factors, together with ion transport systems in a Ca^2+^-dictated manner, the latter of which relates to the four EF-hand domains that force the protein to undergo structural reorganizations upon complexing the ion [137,138]. CaM was suggested to account for the observed Ca^2+^ dependence of CRAC channel inactivation. A stepwise bivalent binding model of CaM to Orai1/Orai3 N-terminal regions was supported by independent studies [139,140]. The propensity of calmodulin to in vitro associate with N-terminal segments of Orai1, the calmodulin-binding domain (CBD_Orai1_), only if Ca^2+^ is present, raised for instance suggestions that CaM was critical for fast inactivation (FCDI) to arise. Although principally consistent with several experimental findings, Ca^2+^-dependent binding of CaM to the relevant Orai1 segment, overall covering the residues 68-91 to underlie FCDI, has been challenged by the crystal structure of dOrai [140,141,142]. This is explained by the fact that the side chains of those residues homologous to W76 and Y80 of human Orai1 proteins that emerged as critical for CaM to associate, reside within the central pathway of ion-permeation, putatively precluding binding of calmodulin for steric reasons [18,140,141,142]. Calmodulin in its Ca^2+^-complexed form was found to associate with the poly-basic domain of STIM1 as well, relying in particular on the residues L390 and F391 of activated STIM1 [83,143,144]. This led to alternative concepts of a role of CaM in slow inactivation (SCDI)rather than FCDI, whereby binding of Ca^2+^-saturated calmodulin was proposed to manipulate the ability of STIM1 to couple to Orai1 and/or the propensity to engage homomeric interactions. Indeed, Li and colleagues reported that overexpression of wildtype calmodulin rather than Ca^2+^-insensitive variants was capable of lessening the level of STIM1–STIM1 association upon store depletion. Considering the residence of _390_LF_391_ in the immediate vicinity of the coupling interface of STIM1 and Orai1, couplings among the CRAC channel and fragments of STIM1 were reported to be sensitive to the presence of Ca^2+^ calmodulin alike, yet awareness is needed that activation of Orai1 channels by STIM1 itself seems to be sensitive to sequence alterations at these two loci as well [83,143]. In any case, interactions of the aforementioned hydrophobic dipeptide and Ca^2+^-bound calmodulin were recently reproduced by Bhardwaj et al., in addition to detecting thereto unseen associations of the Ca^2+^-bound sensor protein with juxtaposed L374 and V375 residues of hSTIM1. Bhardwaj and co-workers revealed that the V375A variant of a basic STIM1 construct disabled of Ca^2+^/calmodulin binding to the lysine-rich element and based on truncations of the polybasic domain, also deprived of SARAF participating in slow inactivation, was alleviated from SCDI [83,143,144]. Contrasting the sensitivity of STIM1-accomplished Orai1 channel activation gating to sequence alterations at the _390_LF_391_ dipeptide, similar patterns were not identified for mutations of the sequentially preceding pair of hydrophobic residues [83,143].

Calsequestrin-1| Calsequestrin-1 (CSQ1) is one of two CSQ isoforms that collectively serve as a main proteinaceous buffer of Ca^2+^ within the sarco/endoplasmic reticulum but differ in terms of expression patterns. Moreover, CSQ proteins perform regulatory functions in Ca^2+^ release from the internal store in particular from the SR and impose inhibitory effects on store-depletion-dependent Ca^2+^ influx [145,146,147,148]. Considering the latter and attempts of Wang et al. that aimed to elucidate CSQ1 function in cell types other than myocytes, CSQ1, also endogenously expressed in HEK293 cells, was shown to associate with STIM1 in a fashion promoted upon store depletion (Figure 2) [145]. The C-terminus of QSQ1 is enriched in acidic residues that are on the one hand responsible for Ca^2+^ sequestration, for protein-interaction-guided anchorage at the ER-membrane close to the Ca^2+^ release machinery if stores are replete, and for associations with the ER-lumen facing span of STIM1 upon store depletion [145,149]. In this regard, it is worth mentioning that CSQ1 is present in a monomeric state at low ER-Ca^2+^ concentrations but engages di- or even polymeric complexes with an increasing filling state of the ER. Consistent with the former findings, CSQ1 was found to preferentially bind to STIM1 in its monomeric state. Furthermore, exposure to an inhibitor of CSQ1 aggregation was identified to interfere to a certain extent with STIM1 redistribution upon store depletion, to suppress STIM1-Orai1 interactions and consequent Ca^2+^ influx [145,146].

TMEM203 and STING| Transmembrane protein 203 (TMEM 203), identified in cDNA overexpression screens of Shambharkar et al. to induce nuclear translocation of Ca^2+^-dependent transcription factors by increasing cytosolic Ca^2+^ levels if expressed at elevated levels, is conserved with regard to vertebrae and predicted to cross the ER membrane with four transmembrane segments. The protein in question was shown to be associated with proteins involved in SOCE, including the IP_3_R release channel, SERCA2, as well as STIM1 (Table 1). Although Shambharkar et al. supposed that TMEM203 overexpression leads to loss of Ca^2+^ from the ER in mouse embryonic fibroblast cells, TMEM203 seems to be also critical for the preservation of ER Ca^2+^ stores, given that siRNA treatment compromised steady-state Ca^2+^ levels of the internal organelle to a significant extent, but this demands further investigation. In addition to this initial study that identified the protein as critical for spermatogenesis and fertility, recently published findings of Li et al. imply that TMEM203 is involved in the regulation of interferon signalling [48]. In this study, the peculiar transmembrane protein was found to regulate the activity of STING (Stimulator of IFN Genes), the latter of which is intricate for the induction of innate immune response to DNA pathogens that rely on IFN production [150,151]. Srikanth et al. showed that a deficiency of STIM1 caused spontaneous activation of the STING pathway, which enhances the expression of type I interferons under resting conditions in mice as well as in a patient with combined immunodeficiency. Coexpression with full-length STIM1 or just a STING-interacting fragment of STIM1 suppressed the function of dominant STING mutants that cause autoinflammatory diseases [151]. Intriguingly, a study by Li et al. was able to establish TMEM203 and STING as capable of binding to one another in a manner occurring in concurrence with the association of STIM1 with STING that impedes spontaneous activation of the latter, in addition to direct interactions between TMEM203 and STIM1. The function of TMEM203 seems to retrieve a high clinical relevance, considering that its transcript levels have been found to be increased in T lymphocytes derived from systemic lupus erythematosus patients [150].

STC2| Zeiger et al. discovered a cell-internal function of the glycoprotein Stanniocalcin 2 (STC2), whereof aberrant expression patterns have been identified in a series of cancer types, in addition to an apparent role thereof in metastasis formation, as a negative modulator of SOCE if residing in the ER lumen that contrasts the well-established endocrine regulation of phosphate as well as Ca^2+^ homeostasis by secreted fish STC. Experimental findings indicated higher levels of Ca^2+^ entry from the extracellular space after store depletion and elevations in intracellular Ca^2+^ in fibroblasts derived from mice ablated from STC2, reproduced in a similar manner upon knock-down in rat H19-7 cells of the hippocampal lineage. Conversely, store-depletion dependent Ca^2+^ influx was markedly attenuated in cells overexpressing STC2. Co-immunoprecipitation indicated that STC2 associates with STIM1 in a fashion insensitive to variations in Ca^2+^ levels and store depletion, showing further a similar affinity to Ca^2+^-insensitive STIM1 (D76A) or the wildtype form, although interactions map to the N-terminus of STIM1 (Figure 2). In agreement with the former, STIM1 translocation and puncta formation were not affected by STC2 couplings either, strongly showing that the regulatory potential on SOCE affects another stage in the signalling cascade [152]. Indeed, subsequent efforts of López and co-workers on regulatory functions of STC2 on SOCE and Ca^2+^ homeostasis in platelets are indicative of alterations in STIM1-Orai1 interactions in the presence and absence of the modulator. Ablation of STC2 was reported to promote Ca^2+^ storage capacity of the ER, aggregation of platelets and Ca^2+^ mobilization upon stimulation with thrombin or treatment with a selective agonist of non-capacitive Ca^2+^ channels [153]. Seemingly in contrast to the findings of Zeiger et al. on murine fibroblasts and hippocampal cells, depletion of STC2 intriguingly correlated with a decrease in STIM1/Orai1 coupling in platelets exposed to thrombin, while STIM1-TRPC1 interactions were promoted [152,153]. Increases in Ca^2+^ influx upon thrombin treatment, the latter of which inherently induces Ca^2+^ entry through store-operated and non-capacitive Ca^2+^ channels, relate rather to non-capacitive channels, considering that Orai3 expression levels, the latter of which may serve as subunit of channels functioning in a non-capacitive nature, was reported to be increased by ~75% in platelets of STC2 knockout mice [153].

Sigma 1 receptor| Sigma 1 receptors (σ1Rs) are integral membrane proteins of the ER and other membranes expressed in various tissues under physiological conditions and are present at elevated levels in several tumours. Considering the ER-associated form, σ1Rs accumulate in subdomains enriched in cholesterol that are associated with mitochondria and interact with BiP, a chaperon present in the ER lumen. If exposed to agonists or upon ER store depletion, σ1Rs are relieved from the former microenvironment and the chaperone, allowing for an interplay with signalling proteins [154,155]. Sigma1 receptors also affect SOCE, yet in view of findings of Gasparre et al. and Srivats and colleagues, implying divergent, cell-type-dependent functional consequences [154,156]. Srivats et al. discovered an interaction between STIM1 and the Sigma1 receptor (σ1Rs) upon co-expression in HEK cells as well as of CHO or MDA-MB-231 cells that endogenously express σ1Rs with agonistic (+)SKF10047 to lead to an inhibition of SOCE (Figure 2). Instead, Ca^2+^ influx was noticed to be enhanced in cells depleted of σ1R or exposed to the antagonist BD1047. The regulator was in this context further found to engage STIM1 within the ER also upon rest, yet in a manner regulated by σ1R activators/inhibitors, respectively. STIM1-bound σ1R proteins are dragged to ER–PM junctions upon store depletion, yet slowing STIM1 translocation and reducing the coupling of activated STIM1 to Orai1, thereby exerting an inhibitory effect on SOCE. In addition, σ1R was observed to pose constraints on store depletion and altogether reduced ER-Ca^2+^ levels [154]. In addition, Brailoiu et al. found that cocaine serves as σ1R agonist as well inhibits SOCE to a considerable extent in endothelial cells isolated from murine brain microvasculature, effects of which may be vanquished upon depletion of the regulator or exposure to antagonists (BD1063 or NE100) [157]. Instead, Gasparre et al. found Ca^2+^ entry after store depletion to be markedly promoted in MCF7σ1 cells that show high expression levels of the specific regulator and SK-N-SH cells originating from neuroblastoma treated with the σ1R agonist (+)-pentazocine, while unaffected in the colon-adenocarcinoma HT-29 lineage and MCF7, the latter of which is a breast-adenocarcinoma derived cell line that shows low densities of σ1R but high levels of σ2R receptor expression. Contrasting with agonist treatment, exposure to antagonistic BD1063 led a considerable ablation in Ca^2+^ influx into MCF7σ1 [156].

Siglec-8| Although mostly neglecting modulatory effects of STIM1/Orai1 post-translational modifications on SOCE herein, Orai1 asparagine-linked glycosylation, occurring exclusively at residue N223 and in a cell-type-specific manner that was recently extensively investigated by Dörr et al., shall be highlighted, given that this may scaffold a binding platform for Siglec-8 (Figure 2). The latter connotes a transmembrane sialic acid-binding lectin protein that preferentially binds to sulfated carbohydrate complexes and comprises immunoglobulin (Ig) like repeats at the extracellular face [158]. Siglec-8 is primarily expressed in mast cells, basophils and eosinophils and seems to have functional relevance to asthma and chronic urticaria, among other mast-cell-associated pathologies [159]. While Siglecs are altogether hardly expressed in the Jurkat T cell line, depletion of otherwise abundant Siglec-8 in HMC1.2 mast cells fostered Ca^2+^ signalling occurring downstream of CRAC channel activation. Thus, Siglec-8 appears to serve as a negative modulator of store-operated Ca^2+^ currents of mast cells, whereas knock-down affects the kinetics of Ca^2+^ influx and allows for heightening in terms of peak-and plateau currents [158].

ORMDL3| ORMDL3 (orosomucoid like 3), encoded by one of three ORMDL genes of the human genome, is a ubiquitously expressed transmembrane protein of the ER [160,161]. As already included in reviews of Kappel et al. and Lopez et al., ORMDL3 limits SOCE in lymphocytes [19,22,160]. ORMDL3 is functionally implicated in slow Ca^2+^ dependent inactivation of CRAC channels by hampering Ca^2+^ permeation across the outermost membrane of mitochondria, hence fostering attenuation of influx currents upon overexpression in HEK293 cells or Jurkat T lymphocytes. Instead, knock-down of ORMDL3 allows for persistent SOCE. The regulatory role of ORMDL3 seems to depend on the N-terminal span of the protein that was identified to co-localize with STIM1 upon rest and after store depletion, whereby both proteins move together to form puncta, yet lacking direct interactions with STIM1 (Figure 2) [160].

CDK1| Smyth et al. showed that the cyclin-dependent kinase 1 (CDK1) interacts with STIM1 (Table 1). During mitosis, SOCE is suppressed independently of changes in expression levels of Orai1 or STIM1. Puncta formation is disrupted, and STIM1 gets phosphorylated in mitotic cells. The authors proposed S486 and S668 as mitosis-specific phosphorylation sites for STIM [49].

### 2.5. Further Interaction Partners of STIM1/Orai1

Apart from protein–protein interactions executing a modulatory function on SOCE, STIM1 and Orai1 have been shown to comprise an affinity to and thereby have the potential to regulate the activity of other proteins implicated in Ca^2+^ signalling.

PMCA| Ritchie et al. revealed that the activation of T-lymphocytes, critically dependent on CRAC channel activation to allow for the activation of Ca^2+^-dependent transcription factors of the nuclear factor of activated T cells (NFAT) family, goes along with downregulations in the PMCA-dependent Ca^2+^-clearance of the cytosol. PMCA1 and PMCA4 were found to be expressed at higher levels as a consequence of T-lymphocyte stimulation. A series of experimental findings postulates a decrease in PMCA function to be dependent on STIM1, the latter of which is expressed at higher levels upon T cell activation. This seems to be mastered via direct interactions between both, and although co-immunoprecipitation efforts indicated that STIM1 binds to PMCA also under resting conditions, fluorescently labelled forms of both were reported to considerably co-localize just after T-cell activation (Table 1). Further, the proline-rich region of STIM1 was shown to be critical for inhibition of PMCA-mediated Ca^2+^ clearance. However, this seems to contribute to the establishment and maintenance of local Ca^2+^ accumulations that are vital for efficient induction of Ca^2+^-dependent downstream responses [50]. Moreover, in aortic smooth muscle cells, Baryshnikov et al. showed that an unperturbed Orai1 function is critical for isoform 1 of the plasma membrane Ca^2+^ pump (PMCA1) and the sodium-calcium exchanger 1 (NCX1) to be expressed at appropriate levels.

Voltage-gated Ca^2+^ channels (CaV1.2)| Cav1.2 is a subunit of L-type voltage-dependent calcium channels (Table 1) [162]. Wang et al. were able to show that STIM1 suppresses Cav1.2 channels whilst activating Orai channels via the SOAR domain of STIM1 [105]. In line with this reciprocal relationship, Park et al. found that STIM1 suppresses the opening of Cav1.2 channels by binding to the C-terminus of Cav1.2, not only inhibiting gating of the channel but also causing a long-term internalization of Cav1.2 channels from the membrane [51]. Further data by Johnson et al. showed that knock-down of Cav1.2 mRNA restored the mitochondrial function in a STIM1-deficient SH-SY5Y cell line [163]. A study by Dionisio et al. indicates that Homer proteins play a role in the communication between STIM1 and Cav1.2 channels [164].

POST (SLC35G1)| The solute carrier family 35 member G1 (SLC35G1), a transmembrane protein located in the ER membrane and the plasma membrane, also termed as transmembrane protein 20 (TMEM20) or partner of STIM1 (POST), respectively, was identified by Krapivinsky et al. Notwithstanding the latter designation, POST binds to Orai1, yet independently of store depletion (Table 1). Upon store depletion, however, POST was reported to bind to STIM1 and to translocate within the ER membrane to reside at ER–PM-junctions. Although these interactions of POST with both fundamental components of the CRAC channel complex inherently lack an effect on SOCE, the protein serves as a negative modulator of energy-consuming transmembrane Ca^2+^ transport. In this regard, store depletion was found to foster binding of the thus formed STIM1–POST complex to sarco/endoplasmic reticulum and plasma membrane Ca^2+^ ATPases (SERCAs and PMCAs, accordingly), yet also to Na/K-ATPase and to nuclear transporter proteins. While the function of POST-mediated STIM1 interactions with importins and exportins remains to be elucidated, the apparent function of POST to ameliorate STIM1 couplings to Ca^2+^ pumps might allow for cytosolic Ca^2+^ elevations to be persistent over longer periods of time, since they hinder these in their activity [52].

AKAP79| Other proteins capable of binding to STIM1 and/or Orai1 serve as scaffold to bring downstream effectors of Ca^2+^ entry into close vicinity of the open channel. This includes the scaffold-constituting protein A-kinase anchoring protein 79 (AKAP79). This protein is suggested to associate with calcineurin and cytosolic NFATc proteins, as well as with Orai1 to form a signalling complex [53]. Thereby, calcineurin and phosphorylated NFATc transcription factors become sequestered within the vicinity of the mouth of CRAC channels. If stores become exhausted after cell stimulation, closeby CaM proteins promote activation and consequent release of NFATc proteins to facilitate nuclear residence [53,165].

ADCY8| Martin et al. identified colocalization of the cAMP-forming enzyme adenylate cyclase 8 (ADCY8), STIM1, and Orai1 in lipid raft domains. Overexpression of Orai1 and STIM1 in HEK293 cells leads to an increase of ADCY8 activity, indicating that Orai1 and STIM1 play an important role in the cAMP-pathway [166]. Another study showed a direct protein–protein interaction between the N-terminus of ADCY8 and the N-terminus of Orai1, which increases after Ca^2+^ store depletion, upregulating the SOCE-induced adenylate cyclase activity in discrete regions of the plasma membrane. These regions are shielded from other calcium events, such as the IP_3_-induced Ca^2+^ release or ionophore-mediated Ca^2+^ entry [54]. Sanchez-Collado et al. reported that overexpression of ADCY8 in breast cancer cells impairs the phosphorylation-dependent Orai1 inactivation [167].

## 3. Conclusions/Outlook

Tight control of cellular Ca^2+^ levels is vital on the single-cell level and further in the hierarchy for the health of the entire organism, as is an efficient entry of the ion upon stimulation. Signalling systems such as the CRAC channel complex that are associated with changes in internal Ca^2+^ conditions are therefore placed into a sophisticated regulatory network with individual proteins exerting a positive or negative effect on store-operated Ca^2+^ entry by modulating plasma membrane-residence of Orai1, scaffolding ER–PM junctions or altogether by sculpturing the ER membrane, allowing for the dynamic establishment of PM microdomains, affecting translocation and cluster formation of STIM1, as well as by promoting or hindering interactions of STIM1 and Orai1 in different respects. Although such regulatory networks are now beginning to emerge, it needs to be emphasized that these might be, at least in parts, obscured in experimental efforts with unphysiological expression levels of STIM1 and Orai1. Though putatively vital under physiological conditions, such accessory proteins are left in disregard in the majority of generically STIM1/Orai1-centered publications. Moreover, inspection thereof demands careful considerations on the respective expression system, given that a set of regulatory mechanisms is apparently cell-type-specific. Altogether, the herein summarized and further modulators of CRAC channel function that remain to be elucidated and/or identified add further complexity to efforts aiming to delineate a holistic view of the complete sequence of events culminating in CRAC channel gating, inactivation, and deactivation. However, knowledge of the entire process of the CRAC channel cascade, including all regulatory proteins involved therein, is of immense importance for the development of future therapeutic targets in the treatment of various human diseases.

## Figures and Tables

**Table 1:** Regulatory proteins within the calcium-release-activated calcium (CRAC) signalling cascade.

Protein	Interaction-Site within STIM1/Orai	Function	References
CCT	Orai1_157–167_	Promotes Orai1 endocytosis	Hodeify et al., 2018 [23]
SNAP-25	not reported	Involved in the increase in cell surface expression of Orai1 upon activation	Woodard et al., 2008 [25]
SPCA2	Orai1	Plasma membrane expression of Orai1, STIM1-independent activation, regulation of SOCE (SPCA2C)	Cross et al., 2013 [32] Smaardijk et al., 2017 [33] Peretti et al., 2019 [34] Feng et al., 2020 [35,36]
Amnionless	Orai1	Endocytosis of Orai1 in the proximal tubulus; critical for albumin re-uptake	Zeng et al., 2017 [37]
RHBDL2	Orai1	Surveillance of Orai1; prevents inappropriate activation and regulates STIM1-Orai1 stoichiometry	Grieve et al., 2020 [38]
Calnexin	STIM1	Plasma membrane expression	Saitoh et al., 2011 [39]
EB1	STIM1_642–645_	Microtubule + end tracking, ER-remodelling, STIM1 motion upon rest	Pozo-Guisado et al., 2013 [40] Sampieri et al., 2009 [20]
APC	STIM1_650–685_	STIM1 clustering at ER-PM junctions	Asanov et al., 2013 [41] Munemitsu et al., 1994 [42]
UBQLN1	Orai1	Promotes Orai1 ubiquitinylation, correlates with a downregulation in CRAC channel activity	Lee et al., 2013 [43]
RTN4	not identified	ER tubulation	Jozsef et al., 2014 [44]
JPH1/2	not identified	ER-PM junctions	Hirata et al., 2006 [45] Woo et al., 2012 [46]
Homer 1a	STIM1	Stabilization of STIM1-Orai1 complex under conditions of rising intracellular Ca^2+^ levels	Jardin et al., 2012 [47]
TM203	STIM1	Regulates interferon signalling (STING-pathway)	Shambharkar, et al., 2015 [48]
CDK1	STIM1	Phosphorylation of STIM1, suppression of SOCE during mitosis	Smyth et al., 2009 [49]
PMCA	STIM1	Suppresses PMCA in its activity upon CRAC channel activation, contributes to the establishment of Ca^2+^ microdomains	Ritchie et al., 2012 [50]
VGCC	STIM1	Inhibits opening of CaV1.2 and leads to withdrawal of CaV1.2 from the plasma membrane	Park et al., 2010 [51]
POST	STIM1/Orai1	negative modulator of energy-consuming transmembrane Ca^2+^ transport	Krapivinsky et al. (2011) [52]
AKAP79	Orai1	Formation of a signalling complex that sequesters downstream acting proteins close to Orai1-formed pores	Kar et al., 2014 [53]
ADCY8	Orai1 N-terminus	Increases in ADCY8 activity upon SOCE at cellular subdomains	Willoughby et al., 2012 [54]

## Data Availability

Not applicable.
